# Fatal visceral disseminated varicella zoster infection during initial remission induction therapy in a patient with lupus nephritis and rheumatoid arthritis—possible association with mycophenolate mofetil and high-dose glucocorticoid therapy: a case report

**DOI:** 10.1186/s13104-018-3271-3

**Published:** 2018-03-05

**Authors:** Masato Habuka, Yoko Wada, Yoichi Kurosawa, Suguru Yamamoto, Yusuke Tani, Riuko Ohashi, Yoichi Ajioka, Masaaki Nakano, Ichiei Narita

**Affiliations:** 1 Division of Clinical Nephrology and Rheumatology, Niigata University Graduate School of Medical and Dental Sciences, 1-757 Asahimachi-dori, Chuo-ku, Niigata City, Niigata 951-8510 Japan; 2 Division of Cellular and Molecular Pathology, Niigata University Graduate School of Medical and Dental Sciences, Niigata, Japan; 3 Pathology and Bioimaging Core Facility, Niigata University Faculty of Medicine, Niigata University, Niigata, Japan; 4 Department of Medical Technology, School of Health Sciences, Faculty of Medicine, Niigata University, Niigata, Japan

**Keywords:** Systemic lupus erythematosus, Lupus nephritis, Mycophenolate mofetil, Glucocorticoid, Disseminated visceral varicella zoster virus infection

## Abstract

**Background:**

Visceral disseminated varicella zoster viral (VZV) infection is a rare but severe complication with a high mortality rate in immunosuppressed individuals, and an increased susceptibility to VZV has been reported in kidney transplant recipients who are treated with mycophenolate mofetil (MMF). In Japan, MMF is currently approved for patients with lupus nephritis (LN) and data to indicate its optimal dosage are still insufficient.

**Case presentation:**

A 46-year-old Japanese woman with rheumatoid arthritis was diagnosed as having systemic lupus erythematosus (SLE) and LN class III (A/C). Although initial remission-induction therapy with prednisolone and tacrolimus was started, her serum creatinine level and urinary protein excretion were elevated. Methylprednisolone pulse therapy was added, and tacrolimus was switched to MMF. Two months after admission when she was taking 40 mg of PSL and 1500 mg of MMF daily, she suddenly developed upper abdominal pain and multiple skin blisters, and disseminated visceral VZV infection was diagnosed. Laboratory examinations demonstrated rapid exacerbation of severe acute liver failure and coagulation abnormalities despite immediate multidisciplinary treatment, and she died of hemorrhagic shock 7 days after the onset of abdominal pain. A serum sample collected at the time of admission revealed that she had recursive VZV infection.

**Conclusions:**

MMF together with high-dose glucocorticoid therapy may increase the risk of VZV infection in Asian patients with SLE. Accumulation of evidence for parameters of safety, such as the area under the blood concentration–time curve of mycophenolic acid, should be urgently considered in order to establish a safer protocol for remission induction therapy in Asian patients with LN.

## Background

Although advances in the application of glucocorticoid and immunosuppressive therapy have vastly improved the prognosis of patients with systemic lupus erythematosus (SLE) over the last few decades [1–3], the occurrence of life-threatening opportunistic infections under such immunosuppressed conditions remains a major complication in such patients.

Mycophenolate mofetil (MMF) has been considered a standard reagent for treatment of diffuse lupus nephritis in Europe and the USA since the late 1990s, although it has been less than a year since MMF was licensed as an immunosuppressant for lupus nephritis in Japan. Visceral disseminated varicella zoster virus (VZV) infection is a very rare complication associated with a high mortality rate in immunocompromised patients [4–10], and increased susceptibility to VZV has been reported in kidney transplant recipients who are treated with MMF [11].

Here we report a rare case of lethal visceral disseminated VZV infection that occurred in a Japanese patient with rheumatoid arthritis (RA) and lupus nephritis (LN) during her first remission induction therapy for LN.

## Case presentation

A 46-year-old Japanese woman with a 9-year history of rheumatoid arthritis, which had been impossible to control even with methotrexate and several biological agents, was referred to our hospital because of leukocytopenia, proteinuria, hypocomplementemia, and positivity for anti-nuclear antibody.

**Fig. 1:**
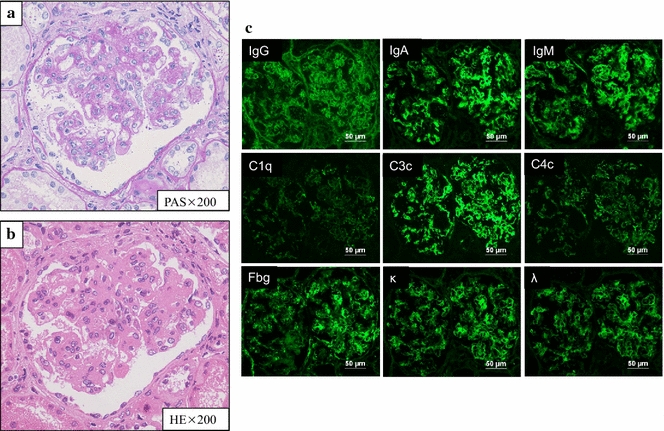
Changes in a kidney biopsy specimen revealed by light and immunofluorescence microscopy. **a**, **b** < 50% of glomeruli displayed segmental or global endocapillary proliferation (**a** periodic acid–Schiff staining) and wire loop lesion (**b** hematoxylin–eosin staining). **c** There was full-house pattern in the mesangial or peripheral capillary loops on immunofluorescence microscopy (positivity for IgG, IgA, IgM, C1q, C3, C4, fibrinogen, kappa and lambda)

On admission, she was 164.5 cm tall and weighed 58.1 kg. She was afebrile, and blood pressure was within normal limits at 104/64 mmHg. Multiple joint swellings with tenderness were evident in the bilateral upper and lower extremities, and pitting edema in her bilateral lower legs. She had been treated with abatacept and methotrexate for her RA. Laboratory examinations demonstrated anemia, lymphocytopenia, positivity for anti-nuclear antibody and anti-DNA antibody, hypocomplementemia, proteinuria, and hematuria (Table 1). A specimen obtained by percutaneous kidney biopsy revealed active and severe lesions such as a massive mesangial matrix and cell proliferations with extensive deposition of immune complexes in mesangial areas and subendothelial lesions. She was then diagnosed as having SLE and LN class III (A/C), according to the ISN/RPS classification criteria (Fig. 1) [12]. Abatacept and methotrexate were discontinued, and the patient was started on initial LN remission-induction therapy with 45 mg of prednisolone (0.8 mg/kg) and 3 mg of tacrolimus daily. This was selected instead of intravenous cyclophosphamide pulse therapy as the patient was unmarried and cyclophosphamide often results in impaired fertility.

**Table 1:** Laboratory data on admission to our hospital

Blood count		
WBC	4160/μL	
Neu	71.1%	
Ba	0.0%	
Eo	1.9%	
Ly	22.4%	
Mo	4.6%	
RBC	350 × 10^4^/μL	
Hb	9.6 g/dL	
Ht	29.8%	
Plt	28.8 × 10^4^/μl	
Serum chemistry		
TP	7.1 g/dL	
Alb	3.0 g/dL	
BUN	16 mg/dL	
Cr	0.51 mg/dL	
UA	4.6 mg/dL	
Na	137 mEq/L	
K	3.7 mEq/L	
Cl	104 mEq/L	
AST	13 IU/L	
ALT	5 IU/L	
LDH	187 IU/L	
ALP	221 IU/L	
TB	0.4 mg/dL	
HbA1c	5.6%	
Urinalysis		
Protein	(1 +)	
Occult blood	(3 +)	
Sugar	(−)	
Urinary sediment		
RBC	50–99/hpf	
WBC	1–4/hpf	
24-h collection		
Protein	0.30 g/day	
Ccr	146 mL/min	
Immunological findings		
CRP	3.44 mg/dL	
IgG	1884 mg/dL	
IgA	595 mg/dL	
IgM	489 mg/dL	
CH50	14 U/mL	
C3	34.5 mg/dL	
C4	6.6 mg/dL	
ANA	640 index	
RF	1439 IU/mL	
ACPA	1058 U/mL	
dsDNA (RIA)	63.7 IU/mL	
SS**-**A	88.1 index	
SS-B	< 5.0 index	
Scl-70	< 5.0 index	
CENPB	< 5.0 index	
Jo-1	< 5.0 index	
LAC	0.95	
CLβ2GPI	13.7 U/mL	
CL-IgG	< 8 U/mL	
MMP3	419.4 ng/mL	
MPO-ANCA	< 1.0	
PR3-ANCA	< 1.0

Her polyarthritis improved immediately, but her urinary protein excretion increased and the serum creatinine level became gradually elevated after the start of therapy. Glucocorticoid pulse therapy followed by 45 mg of prednisolone and 500 mg of MMF daily, instead of tacrolimus, led to gradual improvement of the proteinuria and serum creatinine level. At that time, her peripheral lymphocyte count was almost stable at 920/μl and serum the IgG level was decreased to 670 mg/dl. The dosage of MMF was increased to 1500 mg and prednisolone was tapered by 5 mg every 4 weeks.

**Fig. 2:**
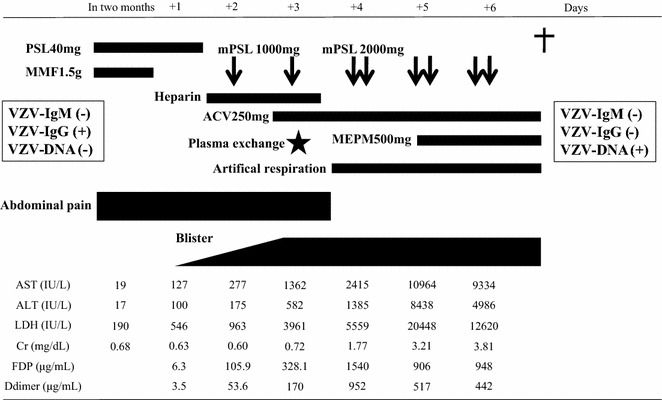
Clinical course of the patient after onset of visceral VZV infection

Two months after admission, the patient complained of upper abdominal pain, and laboratory examinations demonstrated liver dysfunction and coagulation abnormalities. Side effects of MMF were suspected and the agent was withdrawn, but the clinical symptoms and laboratory abnormalities rapidly worsened. Thrombotic microangiopathy associated with SLE was suspected, and additional glucocorticoid pulse therapy together with continuous heparin infusion and plasma exchange were started. Two days later, multiple blisters developed on the patient’s skin and she was diagnosed as having disseminated VZV infection. Despite immediate administration of acyclovir, the liver dysfunction and coagulation abnormalities worsened rapidly along with a severe hemorrhagic tendency. Massive blood transfusion and continuous catecholamine infusion proved ineffective, and the patient died of hemorrhagic shock 7 days after the onset of abdominal pain (Fig. 2).

**Fig. 3:**
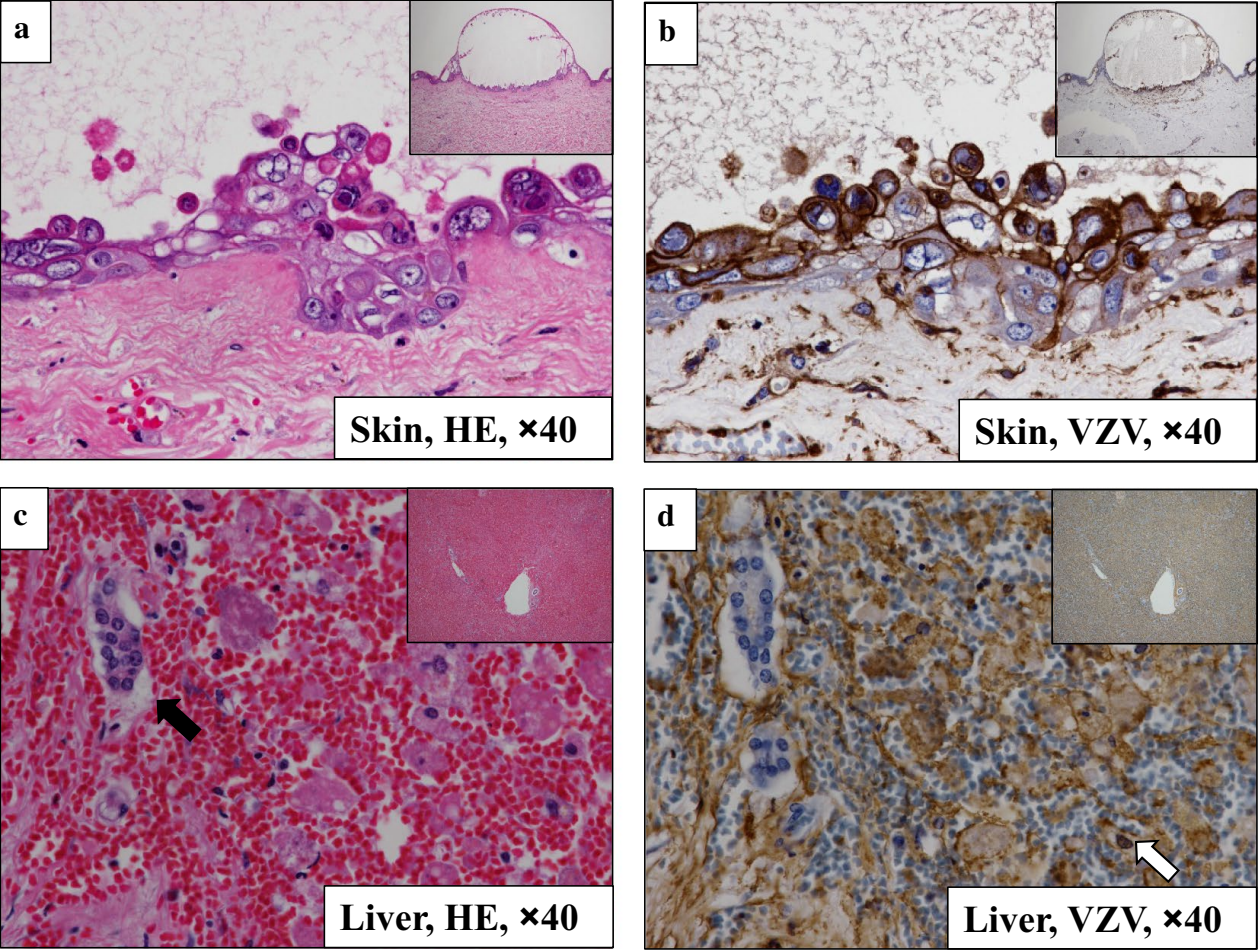
Histological findings at autopsy (**a**, **c** hematoxylin–eosin staining; **b**, **d** immunostaining with anti-VZV antibody). **a**, **b** All of the epidermis, dermis and interstitial tissue of the skin showed positive immunostaining with anti-VZV antibody. Intranuclear inclusion bodies were also identified. **a** Hematoxylin–eosin staining; **b** immunostaining with anti-VZV antibody. Magnification × 40. **c**, **d** Most of the necrotic hepatic tissue and the remaining normal areas were positively immunostained with the anti-VZV antibody, except for the bile ducts (black arrow). Intranuclear inclusion bodies were identified (white arrow). **a** Hematoxylin–eosin staining; **b** immunostaining with anti-VZV antibody. Magnification × 40

At autopsy, the skin lesions showed positive immunostaining with anti-VZV antibody in the epidermis, dermis, and interstitial tissue, and intranuclear inclusion bodies were also identified (Fig. 3a, b). The liver tissue showed severe, extensive necrosis upon hematoxylin–eosin staining and the remaining normal tissue except for the bile ducts showed positive immunostaining with the anti-VZV antibody (Fig. 3c, d). Although a serum sample collected at the time of admission was negative for VZV-IgM and positive for VZV-IgG, another serum sample collected at the time of blister formation was negative for both VZV-IgM and VZV-IgG and positive for VZV-DNA, thus indicating recursive VZV infection after the initiation of LN remission-induction therapy.

## Discussions and conclusions

Visceral disseminated VZV infection, whether due to primary infection or reactivation, is a rare but severe complication with a high mortality rate in immunosuppressed individuals, especially organ transplant recipients [4–10]. Doki et al. reported that visceral VZV infection occurred in 20 (0.8%) of 2411 patients who underwent allogenic hematopoietic stem cell transplantation at a single center in Japan, and the mortality rate was 20% [4]. Although patients with SLE are reported to show a high incidence of herpes zoster [13], disseminated VZV infection as a cause of death is considered to be rare.

In general, lymphopenia is thought to be one of the risk factors for the development of opportunistic infections. Merayo-Chalico et al. considered lymphopenia (< 1000/μl) to be an independent risk factor for the development of severe infections in SLE patients, irrespective of disease activity, and almost one-fourth (23.6%) of SLE patients with severe infections had skin/soft tissue involvement [14]. In addition, previous treatments including biological agents and methotrexate for RA might also have affected the immunocompromised condition of our patient.

MMF has been used as a key drug for severe LN in Europe and the USA [15–17], and a report from China has indicated that MMF for LN patients is more effective and associated with a lower incidence of adverse events, including infections, than oral cyclophosphamide [18]. The recommended dosage of MMF as remission induction therapy for ISN/RPS class III or IV LN is up to 3000 mg daily, together with glucocorticoid pulse therapy and 0.5–1.0 mg/kg prednisolone, according to the guidelines of the American College of Rheumatology (ACR) [15], the European League Against Rheumatism (EULAR) [16], and the Kidney Disease: Improving Global Outcomes (KDIGO) [17], whereas a lower MMF dosage of 1500–2000 mg has been recommended by the Asian Lupus Nephritis Network (ALNN) for Asian LN patients [19].

In Japan, MMF was newly approved as an immunosuppressant for LN in August 2015 by the Ministry of Health, Labour and Welfare. Thereafter, we started to use it as an initial remission induction therapy for 4 patients with LN in our department during the first 6 months, and the patient presented here died of visceral disseminated VZV infection due to reactivation of VZV. Indeed, it is noteworthy that, in our department, we had never previously experienced any such case of severe infection in patients receiving conventional initial remission induction therapy with high-dose glucocorticoid and intravenous pulse cyclophosphamide therapy and/or tacrolimus.

The ALMS study mentioned that serious adverse events, including severe infection, were more prevalent among Asian patients receiving MMF than in other geographical regions [20]. When MMF was introduced as the standard protocol for kidney transplantation after the late 1990s, several reports warned of the association between MMF and increased susceptibility to VZV infection [8, 11]. Moreover, Chakravarty et al. demonstrated that SLE patients had an increased incidence of herpes zoster in comparison to patients with non-inflammatory musculoskeletal conditions, and that the use of prednisone and MMF was an additional risk factor for the infection among SLE patients [13]. Rondaan et al. reported that cellular immunity to VZV was decreased in patients with SLE, whereas it was comparable to that in healthy controls in patients who had granulomatosis with polyangiitis [21].

In our present patient, a relatively high dosage of prednisolone was continued together with MMF. Although currently there is no standard approach for monitoring of adverse events associated with MMF, several reports have indicated that the area under the blood concentration–time curve (AUC) of MPA is a useful parameter for monitoring the therapeutic effect and safety in patients with LN or organ transplantation [22, 23]. MMF is known to have a strong steroid-sparing effect in renal transplant recipients, and the serum concentration of mycophenolic acid (MPA), a metabolic product of MMF, becomes elevated when the dosage of prednisolone is tapered [24]. It is also reported that the MPA AUC has a tendency to increase over time because of its slow metabolic changes, and that there is a progressive decrease in MPA clearance as the glomerular filtration rate declines [25, 26]. Hyunyoung et al. described that high AUC values of MPA in renal transplant recipients were associated with an increased risk of severe infection [27]. Thus, in the present case, continuous high-dose glucocorticoid therapy together with acceleration of the MMF dosage might have led to elevation of the MPA AUC, becoming a cause of lethal VZV infection.

Here we have reported a case of fatal visceral disseminated VZV infection in a Japanese patient with RA receiving glucocorticoid and MMF therapy for LN. Strong immunosuppressive agents have been commonly used in patients with autoimmune disorders, and therefore there is a need for awareness of this severe, unpredictable complication especially when patients are receiving high-dose glucocorticoid in combination with MMF. Although we should recommend patients without IgG anti-VZV antibodies to receive VZV-vaccination before immunosuppressive treatment, there has been no effective method to prevent recursive VZV infection so far. Further accumulation of data such as the relationships between changes in the MPA AUC and adverse events in Asian patients with LN would be of great help for establishing a more appropriate and safer protocol for LN in the near future.

## Abbreviations

AUC: area under the blood concentration–time curve
LN: lupus nephritis
MMF: mycophenolate mofetil
MPA: mycophenolic acid
RA: rheumatoid arthritis
SLE: systemic lupus erythematosus
VZV: visceral varicella zoster virus
